# Heavy Metal Bioaccumulation in Rice from a High Geological Background Area in Guizhou Province, China

**DOI:** 10.3390/ijerph15102281

**Published:** 2018-10-17

**Authors:** Xiangyu Kong, Ting Liu, Ziheng Yu, Zhe Chen, Da Lei, Zhiwei Wang, Hua Zhang, Qiuhua Li, Shanshan Zhang

**Affiliations:** 1 State Key Laboratory of Environmental Geochemistry, Institute of Geochemistry, Chinese Academy of Sciences, Guiyang 550081, China; 18286015062@163.com (X.K.); liuting@ihb.ac.cn (T.L.); ysir20140407@163.com (Z.Y.); chenzhe@glut.edu.cn (Z.C.); leida@mail.gyig.ac.cn (D.L.); 2 Guizhou Provincial Key Laboratory of Mountain Environment Information System and Ecological Environment Protection, Guizhou Normal University, Guiyang 550001, China; 3 Guangxi Key Laboratory of Environmental Pollution Control Theory and Technology, Guilin University of Technology, Guilin 541004, China; 4 Guizhou Institute of Prataculture, Guizhou Academy of Agricultural Sciences, Guiyang 550006, China; wzw1206@163.com; 5 College of Resource and Environmental Engineering, Guizhou University, Guiyang 550025, Guizhou, China; m15519307342@163.com

**Keywords:** high geological background, heavy metals, paddy rice, bioaccumulation

## Abstract

Long-term exposure to high levels of heavy metals can lead to a variety of diseases. In recent years, researchers have paid more attention to mining and smelting areas, industrial areas, and so forth, but they have neglected to report on high geological background areas where heavy metal levels are higher than China’s soil environmental quality standard (GB 15618-2018). In our study, an investigation of heavy metals in paddy soil and rice in the high background area of Guizhou Province was carried out, and the factors affecting the absorption and utilization of heavy metals in rice were discussed. A total of 52 paddy soil and rice samples throughout the high geological background of Guizhou, China, were collected, and concentration(s) of arsenic, cadmium, copper, lead, and zinc were analyzed. The arithmetic mean values of paddy soil heavy metals were 19.7 ± 17.1, 0.577 ± 0.690, 40.5 ± 32.8, 35.5 ± 32.0, and 135 ± 128 mg kg^−1^ for arsenic, cadmium, copper, lead, and zinc, respectively. Most of the heavy metals’ contents in the soil were above the soil standard value. The highest content of cadmium was 15.5 times that of the soil standard value. The concentration(s) of arsenic, cadmium, copper, lead, and zinc in rice were 0.09 ± 0.03, 0.01 ± 0.01, 1.57 ± 0.69, 0.002 ± 0.003, and 11.56 ± 2.61 mg kg^−1^, respectively, which are all lower than those specified by Chinese food safety standards (GB 2762-2017). The results and discussion show that the bioavailability, pH, and soil organic matter are important factors that affect the absorption of heavy metals by rice. According to the consumption of rice in Guizhou Province, the risk of eating rice was considered. The results revealed that the hazard quotient is ranked in the order of copper > zinc > cadmium > arsenic > lead, and there is little risk of eating rice in the high geological background area of Guizhou Province. These findings provide impetus for the revision and improvement of this Chinese soil environmental quality standard.

## 1. Introduction

In recent years, agricultural food safety issues caused by heavy metals and metalloids, including arsenic (As), cadmium (Cd), copper (Cu), lead (Pb), and zinc (Zn) pollution, have become one of the world’s most serious environmental problems [1,2,3,4,5]. Heavy metals are toxic because they tend to accumulate in crops and eventually enter the human body through the food chain, posing a threat to human health. Bioaccumulation results in the concentration(s) of some chemical substances increasing through the food chain and over time. These compounds are absorbed and stored in crops or bodies, compared to the concentration(s) of chemicals in the environment. Heavy metals accumulate in crops rather than being decomposed (metabolized) or excreted from crops [6,7,8]. Different types of toxic heavy metals will cause different degrees of harm to the human body. For example, they may lead to central and peripheral neurological damage, cardiovascular disease, birth defects, placental development disorders, and other diseases [9]. Pb is transmitted through the biological chain to the human body and can cause kidney disease, bone and stomach pain, nerve damage, spontaneous abortion, anemia, and behavioral changes. It has been found in recent research that Pb may alter the life course, as it has a half-life of up to 30 years in bone [10,11,12]. Cd is associated with liver, bone, kidney, and reproductive effects and can cause pain [13,14]. For Cu and Zn, they are essential trace elements in the human body, but when the intake exceeds the safety level, they may also have toxic effects. A large intake of Zn may cause neurotoxicity to humans, and Cu is related to immunotoxicity and developmental toxicity [15].

The excess of heavy metals in soil is the main source of the heavy metals in crops that pose health risks [16]. However, we may have overlooked some practical problems. Previous research showed that the Cd content in peanut seeds is 0.21–0.748 mg kg^−1^ (the maximum value is 1.87 times higher than that specified by China’s food safety standard GB 2762-2017) when the soil Cd content does not exceed the standard (Chinese soil environmental quality standard GB 15618-2018) [17]. When the measured values exceeded the standard and reached 1 to 4 times the food Cd limit standard [18], was there any correlation between the soil being over-standard and crop being over-standard? In other words, when the soil exceeds the standard, does this mean that the crop will also be over-standard or tend to be over-standard?

The southwestern area of China is a typical high geological background (HGB) area. The distribution of heavy metals in the topsoil is mainly attributed to the geological background. Based on the current Chinese soil environmental quality standard (CSEQS) GB 15618-2018, HGB is an important factor leading to excessive heavy metals in soil [19,20,21]. He and other researchers have shown that the soil is formed by the weathering processes of rocks and various sediments. The trace heavy metals contained in the soil are mainly derived from the primary minerals of the rock. Therefore, the composition and content of heavy metals in the rocks determine the heavy metal content in the soil. Heavy metals migrate into the soil in various forms and then are transported through the food chain, which may endanger human health [22,23]. However, levels of heavy metals in the soil exceeding the safety standard that are caused by high geological background are extremely difficult to remediate. Rice is the main food crop in southwestern China. Some researchers have demonstrated that rice is relatively prone to being enriched in heavy metals [24], especially in Cd, Cu, Pb, and As [25,26].

Previous studies have mainly focused on the mining areas, and there has been little research on the background areas, especially the HGB areas where heavy metal levels exceed the Chinese food safety standard (CFSS) and the contents of heavy metals such as As, Cd, Cu, Sb, and Zn in paddy soils exceed the CSEQS [27]. It is essential to explore whether there is a health risk posed by rice grown in high background areas and to reveal the means of bioaccumulation of heavy metals in rice in HGB areas. In this study, the contents of As, Cd, Pb, Cu, and Zn in paddy soils and rice in Guizhou Province were analyzed, and the factors affecting the accumulation and over-standard levels of heavy metals in rice were discussed to support and provide important information for the improvement of soil environmental quality standards in China.

## 2. Materials and Methods

### 2.1. Study Area

The study area is located in Guizhou Province, China (24°37’ N–29°13’ N, 103°36’ E–109°35’ E). This area is 176,167 square kilometers. Guizhou has a population of 35.81 million, and rice is a staple food in this region. Guizhou Province is a karst area with a subtropical humid monsoon climate. The karst landform area accounts for 61.92% of the province. In most areas, the annual average temperature is 14–16 °C and the annual precipitation is generally 1100–1400 mm. The frost-free period lasts approximately 270 days [28,29]. Comparing the background content of heavy metals in Guizhou soil to the background values of the general soil environment in China, it is found that Guizhou Province is a typical HGB region, which belongs to the geological background brought by different parent material weathered soils [30]. The soil texture of the farmland is mostly clay loam. The rice varieties are Indica and Japonica rice. The high background established by different parent materials’ (rock) weathering and the long-term evolution of the paddy soil are important factors affecting the heavy metal content of the paddy soil [27].

### 2.2. Sample Collection and Pretreatment

**Figure 1:**
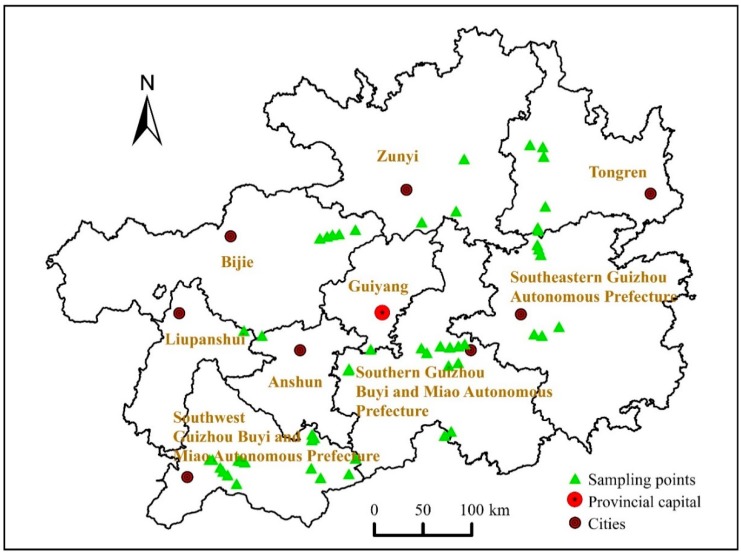
Rice sampling point distribution map in Guizhou Province.

The sample collection of soil and rice avoided all rice fields that are affected by human activities, including densely populated areas and industrial areas: areas of mining and smelting, industrial printing and dyeing, engineering, electroplating, electronics, batteries, chemicals, pesticides, and livestock and poultry farms. The above industries typically produce a variety of wastes and pollutants. During the rice harvesting season, 52 rice samples were collected directly from rice fields in 30 counties of Guizhou Province, and 52 soil samples were collected from rice roots (about 5–10 cm depth). At each sampling site, five subsamples were mixed to obtain one sample, and the samples were taken in duplicate at each site. All rice and soil samples were stored in double consecutive polyethylene bags to avoid cross-contamination and were transferred to the laboratory on the day of sampling (soil samples were stored and transferred with ice packs). The sampling sites are shown in Figure 1.

In the laboratory, two subsamples of wet soil were first homogenized with a blender after removing bigger particles (e.g., stones and plant residues) by passing them first through 50-mesh sieves and subsequently through 200-mesh sieves. The blender was carefully cleaned, first using tap water, then acid-washed, and then rigorously rinsed by ultrapure water and dried by a hair drier before the next sample cycle. Before measurement, each wet soil sample was divided into two, with one subsample used for the determination of heavy metals and the other for water content measurement by drying at 45 °C for 48 h, where the weight of the sample was measured every hour until the weight difference was no more than 5 mg [31]. The rice samples were washed with ultrapure water at least three times and dried at 40 °C to a constant weight. The shell was removed from the seed, then the rice was dehulled to obtain brown rice using a JLGJ-45 electric glutinous rice machine (Taizhou, Zhejiang, China), and the rice bran was taken out by the LTJM-160-type rice cultivating machine (Taizhou, Zhejiang, China). All precautions were taken to avoid any cross-contamination in the process. Prior to the next sample cycle, the mill was rinsed with ultrapure water.

### 2.3. Sample Analysis and Quality Control

Elemental content analysis in rice samples followed the EPA Method 3050B [32]. All samples were ground to fine powder and kept at 4 °C until analysis. For analysis, 0.1000 g of the sample was placed into a Teflon digestion vial with a sensitive balance, using HNO_3_ in the high-temperature and high-pressure closed-condition digestion method [33], and H_2_O_2_ was added after digestion to fully oxidize the organic matter (at 90 °C). The heating acid was heated and diluted to a constant volume, and As, Cd, Cr, Cu, Pb, and Zn were measured by inductively coupled plasma mass spectrometry (ICP-MS, US PE, Nexion 300X). All the reagents used were super-pure or guaranteed reagents, and HNO_3_ was subjected to secondary sub-boiling distillation. The experimental water was ultrapure water. The vessels used in the experiment were all immersed in 20% nitric acid for 48 h and rinsed with ultrapure water 3 times. Three sets of blanks and 10% parallel samples were set aside for each batch of experiments. The Chinese national standards of citrus leaf substance: GBW10020 (GSB-11) and the lobster hepatopancreas reference material for trace metals: TORT-3 were used as reference materials for quality control throughout the analysis process.

The certified and measured concentration(s) of the reference materials and the analytical quality control parameters are shown in Table 1. It can be seen from Table 1 that the recoveries of the six elements of As, Cd, Cr, Cu, Pb, and Zn in the reference materials GBW10020 (GSB-11) and TORT-3 were within the acceptable range. The measurement results were all within the reference value range, and the deviation of the three parallel determinations was less than 10%. These six elements showed good reproducibility for the analysis of parallel samples of each standard material.

### 2.4. Bioaccumulation Factor

The bioaccumulation factor (BAF), i.e., the ratio of the concentration(s) of an element in the grain to that in the corresponding soil, was calculated for each rice sample to quantify the bioaccumulation effect of rice with regard to the uptake of heavy metals from soils [34,35]. The BAF was computed as:

(1) $$\;\mathrm{BAF}=\frac{C_{R}}{C_{S}}\;$$

where *C_R_* (mg kg^−^^1^) and C_S_ (mg kg^−^^1^) represent the concentration(s) of heavy metals in rice grains and soil on a dry-weight basis.

### 2.5. Risk Assessment of Rice Consumption

Among the heavy metals and metalloids of high geological background areas, As, Cd, Cu, Pb, and Zn are usually of high concern. They are prone to accumulate in rice, which has been of great concern around the world [36]. These toxic elements can enter the human body through soil—crop—domestic animals—human pathways or direct human ingestion of soil/dust. Ingestion of heavy metals via rice might be an important exposure pathway as rice is the staple food for the local in habitants.

To assess the overall potential risk posed by all seven heavy metal elements, the hazard index (HI), i.e., the sum of the HQ values of the individual metals, was calculated [37]. The formula for calculating HI is:

(2) $$\;\mathrm{HI}=\sum_{n=1}^{i}{\mathrm{HQ}}_{i}\;$$

where HQ is the health risk assessment of the heavy metal hazard quotient, HQ > 1 indicates potential adverse health effects and HQ > 10 suggests high chronic risk [38]. HQ is generally calculated by the following equation:

(3) $$\;\mathrm{HQ}=\frac{\mathrm{EDI}}{\mathrm{TDI}}\;$$

where EDI and TDI represent estimated daily intake and tolerable daily intake. EDI was calculated as follows:

(4) $$\;\mathrm{EDI}=\frac{R\times \mathrm{HM}}{W}\;$$

where R (g day^−1^), HM (mg kg^−1^) and W (kg) represent the rice consumption, concentration(s) of heavy metals, and average body weight, respectively.

TDI of As, Cd, Cu, Pb, and Zn (50, 1.0, 40, 3.5, and 300 μg kg^−1^ BW, respectively) were calculated from the formula:

(5) $$\;\mathrm{TDI}=\frac{\mathrm{PTWI}}{7}\;$$

where PTWI is the provisional tolerable weekly intake suggested by the Joint FAO/WHO Expert Committee on Food Additives [39,40,41]. BW used in this study was based on questionnaire answers from a survey performed and the U.S. EPA Exposure Factor Handbook for the study area [42]. The mean BW value for adults (18 years old or over) was 59.80 ± 7.19 kg, and the average daily rice intake of adults was considered to be 420 g per day [43].

### 2.6. Statistical Analysis

All data were analyzed using Microsoft Excel 2013 (Microsoft, Redmond, WA, USA), Origin 9.0 (Origin Lab, Northampton, MA, USA), SPSS 13.0 (IBM, Chicago, IL, USA). Contamination levels of heavy metals in soils and rice are expressed as the heavy metal concentration(s) arithmetical mean values and range. Correlation analysis of heavy metal concentration(s) in soil was conducted using the Spearman method. Mann—Whitney and Kruskal—Wallis tests were used for the comparison of three different types of HGB soil heavy metal levels. Both statistics are nonparametric methods, since the heavy metal concentration(s) do not have a normal distribution. Differences were considered to be significant at *p* < 0.05.

## 3. Results and Discussion

### 3.1. Heavy Metal Concentration, pH and SOC in Paddy Soil

In Table 2, the levels of five heavy metals in soil in the study area are presented as the mean ± standard error and are expressed in mg kg^−1^. The arithmetic mean (AM) contents of As, Cd, Cu, Pb, and Zn were 19.7 ± 17.1, 0.577 ± 0.690, 40.5 ± 32.8, 35.5 ± 32.0, and 135 ± 128 mg kg^−1^, respectively. Compared with the soil background level in Guizhou [30], the total concentration(s) of Zn (48.8~907 mg kg^−1^) was significantly elevated, with it being 38.46% higher than the background value, and the upper limit was 9.12 times higher than the background value. Overall, 92.3% of the samples exhibited the total concentration(s) of Cd of less than 1.0 mg kg^−1^, and the median value was 0.416 mg kg^−1^. Kabata-Pendias [44] and Alloway [45] reported that the background Cd concentration(s) (of most of the surface soil on the earth) did not exceed 1.0–1.1 mg kg^−1^, indicating that the concentration(s) of total Cd in Guizhou Province was in the range of geochemical background values. The total concentration(s) of As, Pb, and Cu were comparable to their local background levels throughout the study area. The soil samples with high Zn content also showed a high content of Cu, Cd, and Pb, and there was a very significant correlation between them (r = 0.35~0.504, all *p* < 0.01) (Table 3), indicating that Zn, as well as Cu, Cd, and Pb, had a common source and received substantial replenishment from it (Table 3). The AM of pH and organic matter (OM) was 6.5 + 0.3 and 5.2 + 2.0, respectively. The pH value was lower than the local background value, while the SOC was 1.2 times higher than the background value.

### 3.2. The Heterogeneity of Soil Heavy Metal Content

It can be seen from the variation coefficient of paddy soil in the study area (Table 2) that heavy metals in different regions showed different degrees of variation, in which Cd and Zn exhibited strong variation and the other elements showed moderate variation, with the coefficients of variation with values more than 10. This indicates that heavy metals originate from different parent material (rock). The parent material (rock) was the main influencing factor for the accumulation of heavy metals in large-scale soils [46,47,48,49,50,51,52,53,54,55,56,57,58]. Most of the parent materials in Guizhou Province were limestone and sand shale. The background values of Zn, Pb, As, and Cd in the soil developed by limestone parent material were high, and the background value of Cu in the soil developed by sand shale parent material was high, as well [21,49]. This could be evidence that high content of heavy metals and metalloids in soils is largely a result of HGB, since As and heavy metals are less mobile than other elements. In addition, other anthropogenic activities, such as water irrigation and fertilizer application in agricultural lands, may enhance the accumulation of heavy metals in soils, but it is not significant [50,51], and thus not considered in this study.

### 3.3. Heavy Metal Concentrations in Rice Grains

An overview of heavy metal concentration(s) in rice is presented in Table 4. The total concentration(s) range of As, Cd, Cu, Pb, and Zn in the study area was 0.030–0.192, 0.001–0.099, 0.353–4.373, 0.00003–0.011, and 4.679–21.593 mg kg^−1^, respectively. Arithmetic mean (AM) concentration(s) of total heavy metals in rice grains were ranked as follows: Zn (11.56 ± 2.61) > Cu (1.57 ± 0.69) > As (0.09 ± 0.03) > Cd (0.01 ± 0.01) > Pb (0.002 ± 0.003). Total concentration(s) of As, Cd, Cu, Pb, and Zn at all sites were lower than the Chinese recommended maximum permissible level (MPL).

### 3.4. Regulatory Standards for Heavy Metal Content in Rice

It was found that the As, Cd, and Pb levels of each site were lower than the international MPL. It is interesting that the total concentrations of As, Cd, Cu, Pb, and Zn (AM) in this study were lower than the Chinese and international MPLs. This demonstrates that in the HGB areas with different parent material soil types, the rice heavy metal concentrations are (regardless of the difference in rice varieties) lower than the levels specified by the CFSS.

### 3.5. Health Risk Assessment

**Figure 2:**
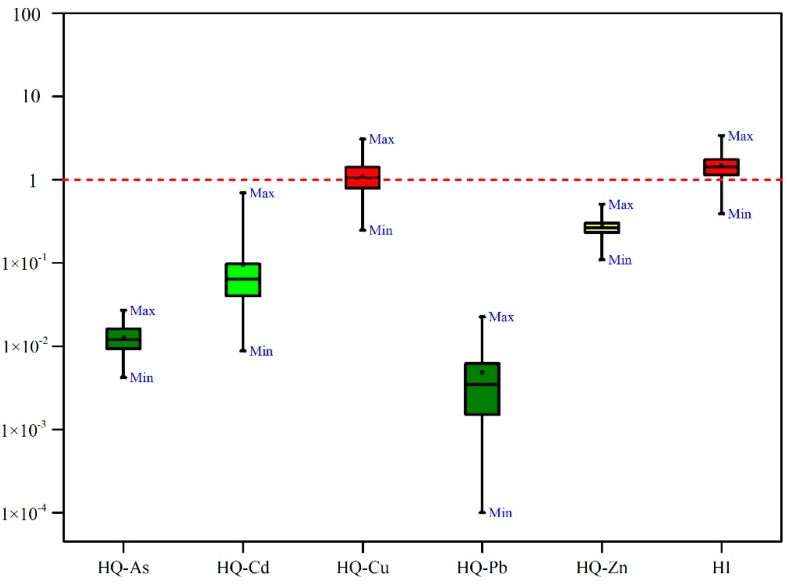
Detailed information of HQs and HI regarding consumption of locally grown rice for adults. **HQs**: Denotes the hazard quotient; HI: Denotes the hazard index.

As the staple food of local residents, rice consumption contributes to an important part of the daily intake of heavy metals. The HQs of individual elements and HI for rice consumption for adults in the HGB area were calculated and are shown in Figure 2. The detailed HQs and HI (with AM, minimum, and maximum) are presented in Table 5.

In the study area, the HQs were ranked in the order of Cu > Zn > Cd > As > Pb. HQ-Cu, HQ-Zn, HQ-Cd, HQ-As, and HQ-Pb accounted for 74.27%, 18.20%, 6.34%, 0.86%, and 0.32% of the HI, respectively. Adults in the HGB area have HQ values greater than 1 for Cu (HQ = 1.10), indicating that Cu absorbed by ingesting rice poses a potential health risk to local residents. The remaining heavy metals have HQ values less than 1. The results show that the health risks posed by As, Cd, Pb, and Zn are negligible.

### 3.6. Risk Consideration

It is essential to evaluate the human health risks caused by rice consumption in the different parent material HGB areas, where heavy metal levels exceed the CSEQS. The results show that in the HGB area of the parent material soil, there is almost no health risk from heavy metals posed by eating locally grown rice. This suggests that in previous studies, we may have overestimated the health risks posed by the HGB formed by the soil’s parent materials.

Rice is the main food in the study area. Although the daily intake of metals or toxic elements through rice is an important pathway for the dietary exposure of local people to heavy metals through food, many studies have reported that human beings are also significantly exposed to metals through other foods such as wheat, vegetables, fruit, fish, meat, eggs, and milk, as well as water [52,53,54]. However, exposure via these foods is rarely considered, so this article has not mentioned it. This paper only provides a reference for the potential health risks caused by the intake of heavy metals or toxic elements in rice grown in the HGB area created by the parent material. In addition, our calculations do not consider special groups such as the elderly, pregnant women, children, and medical patients.

### 3.7. Heavy Metal Bioaccumulation and Influencing Factors

To understand the migration and enrichment of heavy metals in the paddy soil–rice system and the influencing factors, this study introduced the geology and background data of the high background types of the metallogeny belt and alluvial plains to help explore whether these two types also have the same phenomenon as in the HGB created by the soil parent materials. Daye City and Changshu City, both in China, comprise a typical HGB metallogenic belt and alluvial plain [55,56], respectively. The detailed heavy metal concentration(s) (with AM) are presented in Table 6. There were no data for the Zn element in Daye City. The concentration(s) of Cd, Cu, Pb, and As in rice grains grown in the metallogenic belt were 0.04–3.29, 3.10–38.3, 0.08–2.02, and 0.14–1.33 mg kg^−1^, respectively. The Cd, Pb, and As concentrations in rice grains were 2.95, 1.85, and 2.07 times higher than the Chinese suggested MPL, respectively. These results indicated that there was no similar phenomenon of heavy metal absorption by rice grains grown in the metallogenic belt-type HGB areas compared to soil parent material-type HGB areas. The average concentrations of As, Cd, Cu, Pb, and Zn in the HGB region of alluvial plains were lower than those of the Chinese suggested MPL. However, there remained 46 rice samples (29.7% of the total sample number) containing Pb levels in excess of its maximum accepted level in foods. For Cd, the number of samples with similar excessive levels was 1 (0.7% of the total sample number). In all rice grain samples, the highest concentration(s) of Pb and Cd were 0.957 and 0.201 mg kg^−1^, respectively [35]. This demonstrates that the alluvial plain HGB areas did not have the same phenomenon as the soil parent material-type HGB areas.

**Figure 3:**
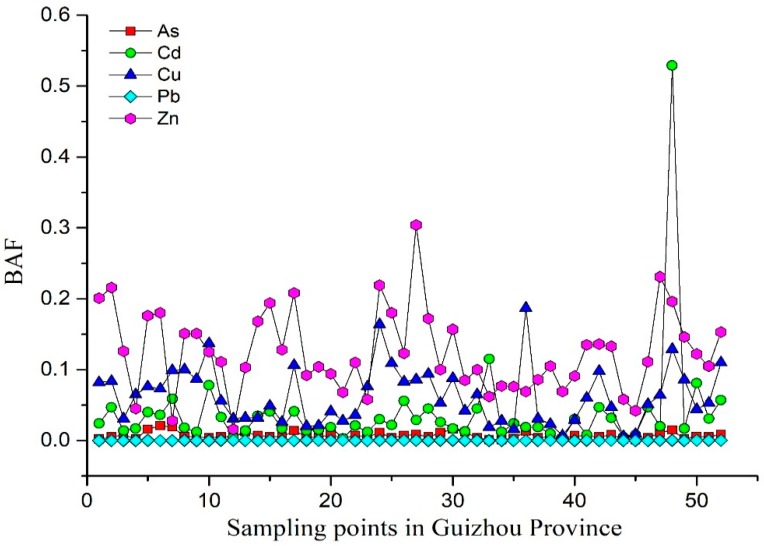
Bioaccumulation of heavy metals in the HGB region of the geotectonic parent type. BAF: Bioaccumulation factor; HGB: high geological background.

**Figure 4:**
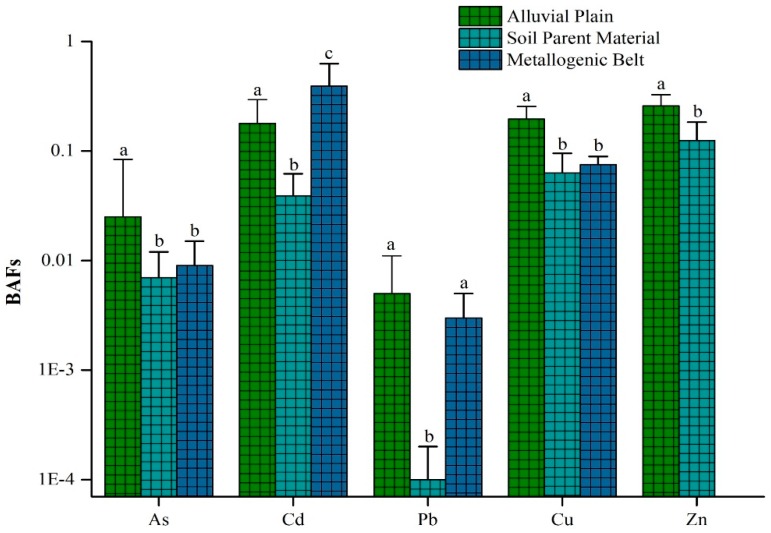
Heavy metal BAFs of rice in different types of HGB areas. Note: The same letter represents no significant difference; the different letters represent a significant difference, *p* < 0.05.

We also calculated the bioaccumulation factor, BAF (BAF = rice heavy metal content/topsoil heavy metal content); BAF is a dimensionless value used to quantitatively analyze the effect of soil heavy metals on crops. The BAF statistical analysis results for each heavy metal from different types of HGB are shown in Figure 3 and Figure 4. There are significant differences in the bioaccumulation of heavy metals in the soils of the three types of HGB regions, which are closely related to the bioavailable heavy metals in their respective soils [57,58,59]. The BAF values of all the heavy metals except for Cd were the highest in the alluvial plain-type HGB zone, as seen in Figure 4. This indicated that the bioavailability of heavy metals in the alluvial plain was the highest. The most important reason for this was that the sediments that carry heavy metals from the higher areas are deposited in the downstream areas; therefore, the heavy metals have been accumulated due to the long-term sediment deposition on the plains.

In HGB areas which originated from different parent materials, all the BAFs of heavy metals were the lowest. Studies have shown that the eutectic substitution of cadmium and calcium often occurs in limestone areas, resulting in strong cadmium stability and low effective cadmium content which is not easily absorbed by plants [60]. Different lithologic regions had complex isomorphic substitutions, which mainly occur in karstic lava areas of Guizhou Province [61]. It is more acceptable that the large-scale karst areas in Guizhou Province are mainly formed by rocks such as limestone, dolomite, marl, and gypsum. They mainly act upon the soil via chemical dissolution and erosion, as well as leaching and collapse; therefore, the free and adsorbed ions of heavy metals in the surface soil are transferred to the underground part, eventually resulting in the decreased bioavailability of heavy metals in the topsoil [62]. Compared to the HGB types of the metallogenic belt and alluvial plain, the heavy metal elements on the surface have no or less related geological effect in the HGB of parent material soil, which explains the reason for the differential heavy metal bioaccumulation in rice grown in the HGB area with the parent material in Guizhou Province compared to in other types of HGB regions.

In addition, soil pH is also an important factor that causes heavy metals to exceed the CSEQS levels. The bioavailability of heavy metals decreases with the increase of soil pH. The lower the soil pH is, the worse the adsorption capacity of soil iron oxides for heavy metals will be [62,63]. In addition, mineral elements can also affect the accumulation and absorption of heavy metals by rice. The higher content of mineral nutrients in soil media, such as Ca, can significantly reduce the rate of Cd absorption in crops, and when these mineral nutrients are lacking in the medium, Cd will be actively transported into the cell through the carrier protein of Ca, due to the similar ionic radius of Cd and Ca [64]. Although the soil medium affects the heavy metal BAF and ability to migrate into the crop under different pH levels, the ion concentration in the soil also changes the enrichment of heavy metals in the crop. Under high-strength fertilization conditions, the ionic strength in the soil is high, and the effectiveness of cadmium is significantly enhanced [60].

In August 2018, China re-released the Chinese Soil Environmental Quality Standard (GB 15618-2018), which classifies heavy metal values into four grades according to the pH of the soil. However, the pH of the soil in China has decreased by 0.13 to 0.80 pH units in recent years, which means that the acidity of the soil has increased by 1.35 to 6.31 times; this extent of acidification takes tens of thousands of years to occur in normal soil processes [65,66]. Our results indicate that the total amount of heavy metals does not necessarily correlate with food security. Studies have shown that the bioavailability of heavy metals in southern paddy soils was more than 60%, and the bioavailability in northern farmland was only about 30% [67]. The proportion of available heavy metals in soils with different geological backgrounds is quite different. Therefore, finding out the soil properties in different regions is a prerequisite for assessing the risk of heavy metals. The current Soil Environmental Quality Standards may not be suitable for the safety risk management of heavy metals in different HGB regions. The phenomenon whereby the soil concentrations are higher than the standard and the rice concentrations are lower than the standard in the HGB area of the parent material type highlights the limitations of the current soil environmental quality standards in China. Incorporating the bioavailability of soil heavy metals into the soil environment quality standard system may be more appropriate and realistic. The relevant soil bioavailability index of soil environmental quality standards in Japan has a certain reference value [68].

## 4. Conclusions

In the HGB area of the parent material type in Guizhou Province, China, eating locally grown rice is the main factor for human exposure to toxic heavy metals. The heavy metal content in the paddy soil exceeds the acceptable levels as stated by the CSQES, but the heavy metal content of the rice does not exceed those of the CFSS. Except for the potential human health risks of Cu, risks from other heavy metal elements do not exist at present. The bioaccumulation of heavy metals in soil is characterized by the presence of different weathering minerals in the HGB area of Guizhou and the unique karst characteristics, which induce high levels of the heavy metal elements on the surface soil, but the levels of bioavailable heavy metals that can be absorbed and utilized by rice are low.

To ensure the quality and safety of soil and agricultural products, and to promote the revision and improvement of soil environmental quality standards, the following is proposed: a simple evaluation system based on the total amount of heavy metals may not be applicable to high geological background areas. It is suggested that a comprehensive evaluation of relevant parameters be formulated, such as the proportion of bioavailable heavy metals, pH values, and levels of organic matter in the soil nationwide, and that limiting values for heavy metal classification in different regions be established.

## Figures and Tables

**Table 1:** Metal concentration(s) identified and measured in the reference materials of GBW10020 (GSB-11) and TORT-3 (mean ± SD; mg/kg) and the recovery (%).

	Measured Value (mg kg^−1^)		Certified Value (mg kg^−1^)		Recovery (%)	
Elements	GSB-11	TORT-3	GSB-11	TORT-3	GSB-11	TORT-3
Cr	1.26 ± 0.13	2.08 ± 0.08	1.25 ± 0.11	1.95 ± 0.24	100.80	106.67
Cu	5.57 ± 0.23	478.64 ± 6.43	6.60 ± 0.50	497 ± 22	94.39	96.18
Zn	15.19 ± 0.63	118.56 ± 1.69	18 ± 2	136 ± 6	94.39	87.18
As	1.12 ± 0.04	64.55 ± 0.54	1.10 ± 0.20	59.5 ± 3.8	101.82	108.49
Cd	0.19 ± 0	39.35 ± 0.34	0.17 ± 0.02	42.3 ± 1.8	111.76	93.03
Pb	10.03 ± 0.23	0.19 ± 0	9.70 ± 0.90	0.225 ± 0.018	103.40	84.44

**Table 2:** Heavy metal concentration(s), pH and OM in paddy soil (mg kg^−1^).

Elements	AM	Maximum	Minimum	CV (%)	Median	Standard Value ^a^	Background Value ^b^
As	19.7 ± 17.1	129	1.08	76.65	15.1	30	20
Cd	0.58 ± 0.69	6.23	0.16	151.93	0.42	0.4	0.659
Cu	40.5 ± 32.8	158	4.03	81.90	29.1	50	32
Pb	35.5 ± 32.0	290	12.4	57.34	27	100	35.2
Zn	135 ± 128	907	48.8	106.34	94.5	200	99.5
pH	6.5 ± 0.3	7.1	5.6	5.1	6.5	null	6.2
OM	5.2 ± 2.0	10.3	1.7	38.5	4.8	null	4.3

^a^ From provisions on the limits of heavy metals in agricultural lands in the Soil Environmental Quality Standards of the People’s Republic of China (GB 15618-2018); ^b^ The soil background value of Guizhou Province comes from CNEMC (1990).; C V: Denotes the coefficient of variation; AM: Denotes the arithmetic mean; OM: organic matter.

**Table 3:** Spearman’s correlations of heavy metals in soils (*n* = 52).

Elements	Pb	As	Cd	Zn	Cu
Pb	1	0.188	0.287 *	0.350 **	0.005
As		1	0.198	0.23	0.282 *
Cd			1	0.476 **	0.259
Zn				1	0.504 **
Cu					1

* Significantly correlated at the 0.05 level (both sides), ** Significantly correlated at the 0.01 level (both sides).

**Table 4:** Heavy metal content and standard limits of rice grains (mg kg^−1^).

Elements	Sample	AM	Maximum	Minimum	CV (%)	Chinese Standardized Value ^a^	International Standardized Value ^b^
As	52	0.09 ± 0.03	0.192	0.03	33.33	0.5	0.2
Cd	52	0.01 ± 0.01	0.099	0.001	100	0.2	0.4
Cu	52	1.57 ± 0.69	4.373	0.353	43.95	10	Null
Pb	52	0.002 ± 0.003	0.011	0.0003	150	0.2	0.2
Zn	52	11.56 ± 2.61	21.593	4.679	22.58	50	Null

^a^ From provisions on the limitation of heavy metals in rice grains in the National Food Safety Standard of the People’s Republic of China (GB 2762-2017) and the Agricultural Industry Standards of the People’s Republic of China (NY 861-2004); ^b^ From the Codex Alimentarius Commission (CAC) Standard Codex Stan 193-1995: adopted in 1995; revised in 1997, 2006, 2008, and 2009; amended in 2010, 2012, 2013, and 2014.

**Table 5:** Detailed information of HQs and HI regarding consumption of locally grown rice for adults.

Types	HQ-As	HQ-Cd	HQ-Cu	HQ-Pb	HQ-Zn	HI
AM	0.0128	0.0943	1.1045	0.0048	0.2707	1.4870
MIN	0.0042	0.0088	0.2482	0.0001	0.1095	0.3920
MAX	0.0269	0.6939	3.0712	0.0225	0.5055	3.3792
Median	0.0120	0.0642	1.0417	0.0035	0.2633	1.4234

**Table 6:** Heavy metal concentration(s) in soil and rice of alluvial plain and metallogenic belt type HGB (mg kg^−1^).

	Alluvial Plain (*n* = 155)		Metallogenic Belt (*n* = 70)	
Elements	Soil	Rice	Soil	Rice
As	8.60 ± 1.93	0.199 ± 0.114	35.4 ± 15.6	0.32 ± 0.09
Cd	0.168 ± 0.181	0.019 ± 0.021	2.59 ± 2.89	1.02 ± 0.67
Cu	44.5 ± 22.9	0.171 ± 0.126	120 ± 47.5	0.36 ± 0.10
Pb	30.5 ± 18.0	3.84 ± 1.07	386 ± 587	28.95 ± 8.22
Zn	90.1 ± 46.4	19.1 ± 3.26	null	null

## References

[1] Chen Y., Huang Y., Zhang W. (2013). Distribution characteristics of soil heavy metal pollution in the lead-zinc mine of Qinglin Township, Xiangyan Town, Pingwu County, Sichuan Province. Acta Miner. Sin..

[2] Jacob J.M., Karthik C., Saratale R.G., Kumar S.S., Prabakar D., Kadirvelu K., Pugazhendhi A. (2018). Biological approaches to tackle heavy metal pollution: A survey of literature. J. Environ. Manag..

[3] Wu W., Wu P., Yang F., Sun D.L., Zhang D.X., Zhou Y.K. (2018). Assessment of heavy metal pollution and human health risks in urban soils around an electronics manufacturing facility. Sci. Total Environ..

[4] Duan H., Hu J., Tan Q., Liu L., Wang Y., Li J. (2016). Systematic characterization of generation and management of e-waste in China Environ. Sci. Pollut. Res. Int..

[5] Duan J., Lee Y., Liu H., Chen H. (2016). Hu Distribution of heavy metal pollution in surface soil samples in China: A graphical review Bull. Environ. Contam. Toxicol..

[6] Lenntech (2016). Heavy Metals. http://www.lenntech.com/processes/heavy/heavy-metals/heavy-metals.html.

[7] Lang W. (2010). Environmental Geochemical Behavior of Heavy Metals Such as Cd in Typical Soils of the Yangtze River System and Watershed. Ph.D. Thesis.

[8] Arora K., Sharma S. (2011). Bioremediation of Heavy Metals. Lap Lambert Acad..

[9] Jomova K., Jenisova Z., Feszterova M., Baros S., Liska J., Hudecova D., Rhodes C.J., Valkoc M. (2011). Arsenic: Toxicity, oxidative stress and human disease. J. Appl. Toxicol..

[10] Anjos J.A.S.A., Sánchez L.E. (2001). Plano de gestão ambiental para sítios contaminados por resíduos industriais-o caso da Plumbum em Santo Amaro da Purificação/BA. Rev. Bahia Análises Dados.

[11] Obeng-Gyasi E. (2018). Lead Exposure and Oxidative Stress—A Life Course Approach in US Adults. Toxics.

[12] Muller C., Sampson R.J., Winter A.S. (2018). Environmental Inequality: The Social Causes and Consequences of Lead Exposure. Ann. Rev. Sociol..

[13] Shaheen N., Ahmed M.K., Islam M.S., Habibullah-Al-Mamun M., Tukun A.B., Islam S., Rahim A.T.M.A. (2016). Health risk assessment of trace elements via dietary intake of non-piscine protein source foodstuffs (meat, milk and egg) in Bangladesh Environ. Sci. Pollut. Res..

[14] Bosch A.C., O’Neill B., Sigge G.O., Kerwath S.E., Hoffman L.C. (2016). Heavy metals inmarine fish meat and consumer health: A review. J. Sci. Food Agric..

[15] Agency for Toxic Substances and Disease Registry (ATSDR) (2005). Toxicological Profile for Zinc.

[16] Wang X.N., Gu Y.G., Wang Z.H. (2018). Biological risk assessment of heavy metals in sediments and health risk assessment in bivalve mollusks from Kaozhouyang Bay, South China. Mar. Pollut. Bull..

[17] Xia S., Wang X., Su G. (2015). Effects of drought on cadmium accumulation in peanuts grown in a contaminated calcareous soil. Environ. Sci. Pollut. Res..

[18] Wang S.S., Wang Y.H., Zhang H. (2007). Cd-Contaminating Peanut Seeds: Distribution Characte risticsof Cadmium and Risk Assessment on Dietary Health. J. Agro-Environ. Sci..

[19] https://img1.17img.cn/17img/files/201807/ueattachment/b13d2dfe-f1e4-4d97-9b42-a39ec3435638.pdf.

[20] Pope G.A. (2015). Chapter 4-Regolith and Weathering (Rock Decay) in the Critical Zone. Dev. Earth Surf. Process..

[21] Huang H.Q., He T.B., Yan L. (2016). Effects of parent rock (parent material) on soil type and distribution in Guizhou. Zhejiang Agric. Sci..

[22] He T.B., Dong L.L., Liu Y.S. (2006). Study on soil physical and chemical properties and heavy metal content in different parent materials in Wudang District, Guiyang City. J. Soil Water Conserv..

[23] Lu S.F. (2015). Assessment of Sediment Pollution and Heavy Metal Enrichment in Harbin Section of Songhua River. Ph.D. Thesis.

[24] Xiao L., Guan D., Peart M.R. (2017). The influence of bioavailable heavy metals and microbial parameters of soil on the metal accumulation in rice grain. Chemosphere.

[25] McBride M.B. (2003). Toxic metals in sewage sludge-amended soils: Has promotion of beneficial use discounted the risks?. Adv. Environ. Res..

[26] Liu H.Y., Probst A., Liao B.H. (2005). Metal contamination of soils and crops affected by the Chenzhou lead/zinc mine spill (Hunan, China). Sci. Total Environ..

[27] Kong X.Y., Huang G.P., Cheng T.J., Jiang F., Wang Z.W., Li Q.H., Yu Z.H., Zhang H., Yao H. (2018). Distribution characteristics of heavy metals in paddy soils in Guizhou Province. Bull. Mineral. Petrol. Geochem..

[28] Ji Y.B. (2006). Status and Analysis of Cadmium Pollution in Agricultural Soils in Guizhou Province. Ph.D. Thesis.

[29] Deng X.H., Bi K. (2004). Analysis of Distribution Area and Distribution Characteristics of Karst Landforms in Guizhou Province. Guizhou Geol..

[30] China National Environmental Monitoring Center (CNEMC) (1990). The Backgrounds of Soil Environment in China.

[31] State Pharmacopoeia Commission (2015). Pharmacopoeia of the People’s Republic of China.

[32] USEPA (1996). The SW-846 Compendium of US Environmental Protection Agency. https://www.epa.gov/hw-sw846/sw-846-compendium.

[33] Liang Q., Jing H., Gregoire D.C. (2000). Determination of trace elements in granites by inductively coupled plasma mass spectrometry. Talanta.

[34] Hang X., Wang H., Zhou J. (2009). Risk assessment of potentially toxic element pollution in soils and rice (*Oryza sativa*) in a typical area of the Yangtze River Delta. Environ. Pollut..

[35] Zhang H., Feng X.B., Larssen T. (2010). Bioaccumulation of methylmercury versus inorganic mercury in rice (*Oryza sativa* L.) grain. Environ. Sci. Technol..

[36] Zavala Y.J., Duxbury J.M. (2008). Arsenic in rice: I. Estimating normal levels of total 19 arsenic in rice grain. Environ. Sci. Technol..

[37] Hough R.L., Breward N., Young S.D., Crout N.M.J., Tye A.M., Moir A.M., Thornton I. (2004). Assessing potential risk of heavy metal exposure from consumption of home-produced vegetables by urban populations. Environ. Health Perspect..

[38] Leung A.O.W., Duzgoren-Aydin N.S., Cheung K.C., Wong M.H. (2008). Heavy metals concentration(s)s of surface dust from e-waste recycling and its human health implications in southeast China. Environ. Sci. Technol..

[39] UNEP/FAO/WHO (1992). Assessment of Dietary Intake of Chemical Contaminants.

[40] World Health Organization (WHO) (1993). Evaluation of Certain Food Additives and Contaminants (41st Report of the Joint FAO/WHO Expert Committee on Food Additives).

[41] US-EPA, IRIS United States, Environmental Protection Agency, Integrated Risk Information System. http://cfpub.epa.gov/ncea/iris/index.cfm?fuseaction=iris.showSubstanceList.

[42] USEPA (1996). Exposure Factors Handbook. Volume I General Factors.

[43] Wu D., Yang X.Z., Li C.X. (2013). Evaluation of Heavy Metal Contents and Health Risks in Rice Soil and Rice in Typical Lead-Zinc Mining Areas of Guizhou. J. Agro-Environ. Sci..

[44] Kabata-Pendias A. (2000). Trace Elements in Soils and Plants.

[45] Alloway B.J., Alloway B.J. (1995). Cadmium. Heavy Metals in Soils.

[46] Xu H.Q., Huang Y.H., Wu Z.F. (2016). As and Cd pollution of agricultural soils in Guangzhou and their multi-scale responses to landscape heterogeneity. Chin. J. Appl. Ecol..

[47] Luque-Espinar J.A., Pardo-Igúzquiza E., Grima-Olmedo J. (2018). Multiscale analysis of the spatial variability of heavy metals and organic matter in soils and groundwater across Spain. J. Hydrol..

[48] Nanos N., Martín J.A.R. (2012). Multiscale analysis of heavy metal contents in soils: Spatial variability in the Duero river basin (Spain). Geoderma.

[49] Fang C.H., Fu Y.Z. (1992). Research on the Compilation of Soil Environment Background Value Map in Guizhou Province. China Environ. Monit..

[50] Ignatowicz K. (2008). Pesticide and Heavy Metals Concentration(s)s in Natural Water near Graveyard in Podlasie Region. https://www.researchgate.net/publication/282675231_Pesticide_and_heavy_metals_concentrations_in_natural_water_near_graveyard_in_podlasie_region.

[51] Wang X., Liu W., Li Z., Teng Y., Christie P., Luo Y. (2017). Effects of Long-Term Fertilizer Applications on Peanut Yield and Quality and on Plant and Soil Heavy Metal Accumulation. Pedosphere.

[52] Chary N.S., Kamala C.T., Raj D.S.S. (2008). Assessing risk of heavy metals from consuming food grown on sewage irrigated soils and food chain transfer. Ecotoxicol. Environ. Saf..

[53] Sipter E., Rozsa E., Gruiz K., Tatrai E., Morvai V. (2008). Site-specific risk assessment in contaminated vegetable gardens. Chemosphere.

[54] Wang X.L., Sato T., Xing B.S., Tao S. (2005). Health risks of heavy metals to the general public in Tianjin, China via consumption of vegetables and fish. Sci. Total Environ..

[55] Zhang L.M., Yu D.S., Shi X.Z. (2012). Simulation soil organic carbon change in China’s Tai-Lake paddy soils. Soil Tillage Res..

[56] Shi X., Fang R., Wu J. (2012). Sustainable development and utilization of groundwater resources considering land subsidence in Suzhou, China. Eng. Geol..

[57] Zhao K., Liu X., Xu J., Selim H.M. (2010). Heavy metal contaminations in a soilerice system: Identification of spatial dependence in relation to soil properties of paddy fields. J. Hazard. Mater..

[58] Zeng F., Ali S., Zhang H., Ouyang Y., Qiu B., Wu F., Zhang G. (2011). The influence of pH and organic matter content in paddy soil on heavy metal availability and their uptake by rice plants. Environ. Pollut..

[59] Monterroso C., Rodríguez F., Chaves R., Diez J., Becerra-Castro C., Kidd P.S., Macías F. (2014). Heavy metal distribution in mine-soils and plants growing in a Pb/Zn-mining area in NW Spain. Appl. Geochem..

[60] Baize D., van Oort F. (2014). Potentially Harmful Elements in Forest Soils. PHEs, Environment and Human Health.

[61] Chen N.C., Zheng R.I., Lei S.R. (2015). Analysis of the Causes of Heavy Metals Exceeding Standards in Agricultural Products. Qual. Saf. Agric. Prod..

[62] Liu Z.G. (2016). Study on Geotechnical Investigation and Foundation Treatment of Karst Foundation. Resour. Inf. Eng..

[63] Yin L.P., Zhang B., Li A. (2014). Effects of soil pH on the behavior of heavy metals in soil. Liaoning Chem. Ind..

[64] Reeves P.G., Chaney R.L. (2008). Bioavailability as an issue in risk assessment and management of food cadmium: A review. Sci. Total Environ..

[65] Guo J.H., Liu X.J., Han W.X. (2010). Significant Acidification in Major Chinese Croplands. Science.

[66] Zhu Q., De V.W., Liu X. (2018). Enhanced acidification in Chinese croplands as derived from element budgets in the period 1980–2010. Sci. Total Environ..

[67] Xu W.H., Guo S.H., Hu Y.M. (2007). Re-evaluation of heavy metal pollution and morphological analysis of cadmium in Zhangshi Irrigation District of Shenyang. Chin. J. Appl. Ecol..

[68] Chen P. (2014). Current Status and Enlightenment of Soil Environmental Quality Standard System in Japan. Environ. Sustain. Dev..

